# Interstitial lung disease associated with inflammatory myositis: Autoantibodies, clinical phenotypes, and progressive fibrosis

**DOI:** 10.3389/fmed.2023.1068402

**Published:** 2023-03-16

**Authors:** Angela Ceribelli, Antonio Tonutti, Natasa Isailovic, Maria De Santis, Carlo Selmi

**Affiliations:** ^1^Department of Rheumatology and Clinical Immunology, IRCCS Humanitas Research Hospital, Milan, Italy; ^2^Department of Biomedical Sciences, Humanitas University, Milan, Italy; ^3^IRCCS Humanitas Research Hospital, Milan, Italy

**Keywords:** antisynthetase, autoantibodies, progressive pulmonary fibrosis, antinuclear antibodies, idiopathic inflammatory myopathy, connective tissue disease

## Abstract

Progressive pulmonary fibrosis is generally diagnosed when interstitial lung disease progression occurs in the absence of any other cause, and a subset of patients with myositis and associated interstitial lung disease may develop progressive pulmonary fibrosis. Numerous autoantibodies (e.g., against tRNA-synthetase, MDA5, Ro52) increase the risk of this clinical feature in myositis and we speculate that serum biomarkers, sought using the most sensitive laboratory techniques available (i.e., immunoprecipitation) may predict pulmonary involvement and allow the early identification of progressive pulmonary fibrosis. We herein provide a narrative review of the literature and also present original data on pulmonary fibrosis in a cohort of patients with myositis and serum anti-Ro52 with interstitial lung disease. Our results fit into the previous evidence and support the association between anti-Ro52 and signs of pulmonary fibrosis in patients with inflammatory myositis. We believe that the combination of available and real-life data has significant clinical relevance as a paradigm of serum autoantibodies that prove useful in determining precision medicine in rare connective tissue diseases.

## 1. Introduction

Idiopathic inflammatory myopathies include clinical subtypes represented by dermatomyositis (DM), polymyositis (PM), immune necrotizing myositis, antisynthetase syndrome (ASSD), and inclusion-body myopathy. This is a spectrum of chronic inflammatory and autoimmune conditions characterized by variable clinical and immunological features (1), such as the prominent skin involvement or the vasculitis in DM (2), the coexistence of Raynaud phenomenon, arthritis, muscle damage, and interstitial lung disease (ILD) in ASSD (3), features that are generally absent in the immune necrotizing or inclusion-body myopathies (4, 5). Whether patients diagnosed with PM should be regarded as a separate group or rather included in the others remains a topic for debate (6).

There have been reports of a growing number of myositis-specific (MSA) and myositis-associated (MAA) autoantibodies in different conditions to predict organ involvement and comorbidities. While MSA are found almost uniquely in patients with idiopathic inflammatory myopathies, MAA are also observed in other connective tissue diseases such as systemic sclerosis, systemic lupus erythematosus, or Sjögren’s syndrome (7). Based on their specific nature and the observation that their coexistence is virtually exceptional, MSA have been proposed to become major determinants for the taxonomy of idiopathic inflammatory myopathies (8) and different specificities can help stratifying patients into groups with homogenous phenotypes (6, 9). As an example, DM with positive anti-Mi-2 antibodies is associated with severe muscle involvement (10), whereas anti-MDA5 antibodies positivity is associated with clinically amyopathic DM, peculiar skin features, and rapidly-progressive ILD (11).

While idiopathic inflammatory myopathies represent less than 5% cases of ILD observed by pulmonologists (12), the prevalence of ILD has been estimated as 40% in idiopathic inflammatory myopathies, reaching highest prevalence rates in ASSD and in clinically amyopathic DM (13, 14) where it is associated with significant morbidity and mortality (15, 16). As we are going to describe in the present review, the risk of developing ILD, its phenotype and progression vary significantly in different idiopathic inflammatory myopathies (17) and an adequate identification of MSA and MAA is expected to predict ILD onset and outcome.

## 2. Progressive pulmonary fibrosis in rheumatology

The concept of progressive pulmonary fibrosis (PPF) has been introduced to indicate every fibrosing ILD other than idiopathic pulmonary fibrosis which demonstrates clinical and/or radiological and/or functional signs of progression with no primitive explanation (18). It has been estimated that up to 40% of ILD cases other than idiopathic pulmonary fibrosis evolve into a PPF phenotype (19). While the incidence of progressive fibrosis in patients with idiopathic inflammatory myopathy-ILD remains unclear (20), there are reports suggesting that a considerable proportion of subjects may evolve to PPF during the disease course in the presence of established risk factors (15, 21) such as older age, extensive fibrosis at high-resolution computed tomography (HRCT) (i.e., traction bronchiectasis, usual interstitial pneumonia – UIP pattern), progression or non-stabilization with initial therapy, and short telomere syndromes (22). Fibrotic HRCT pattern at baseline, diabetes mellitus and steroid-use have been identified as risk factors for PPF in patients with connective tissue disease-ILD (23). Short disease course, African American ethnicity, and gastro-esophageal reflux are considered specific risk factors for PPF in patients with systemic sclerosis-ILD, whereas the smoking status is associated with PPF in rheumatoid arthritis-associated ILD, and the extension of lung involvement at HRCT is a risk factor in both systemic sclerosis-ILD and rheumatoid arthritis-ILD (22). The results of the SENSCIS and INBUILD trials have shown that nintedanib is an antifibrotic treatment that leads to significant reduction in forced vital capacity 1-year decline in patients with systemic sclerosis-ILD and progressive fibrosing ILD (24, 25).

Older age, reduced forced vital capacity, ground-glass opacities, acute and subacute onset, and extent of abnormalities at HRCT represent unfavorable prognostic factors for idiopathic inflammatory myopathy-associated ILD in a meta-analysis by Kamiya and Colleagues; in the same report, anti-Jo-1 antibody was associated with favorable outcomes (26) but the authors admitted the low quality of supporting data. When considering only ASSD, features such as signs of fibrosis at HRCT, smoking status, and lung damage biomarkers (such as surfactant protein D) have been associated with worse outcomes (21, 27). However, these studies evaluated the prognosis of idiopathic inflammatory myopathy-ILD without distinguishing specific clinical, functional, and radiological trajectories. Taken altogether, the lines of evidence demonstrate no established risk factors for PPF in patients with idiopathic inflammatory myopathy-ILD, and the proportion of these patients undergoing PPF remains largely unknown.

## 3. Autoantibodies in idiopathic inflammatory myopathy-ILD at risk for progressive pulmonary fibrosis

Myositis autoantibodies are ideal candidates for precision medicine, being associated with clinical features and prognosis with one of the highest degrees of specificity among serum autoantibodies, as also demonstrated in ILD patients (28). Table 1 summarizes the major elements of the association between myositis autoantibodies and ILD (11, 28–50). Chronic, insidious, non-specific interstitial pneumonia (NSIP) with extensive ground glass opacity is the most common manifestation of ILD in patients with ASSD (29), especially when combined with organizing pneumonia; however, a UIP pattern can be observed in up to 10% cases and is associated with PPF (3, 30). The risk of ILD in patients with ASSD is highest with anti-PL-7, anti-PL-12, and anti-EJ antibodies (31). However, ILD is the leading cause of mortality in ASSD, independent of the serologic status (28), as for the anti-MDA5 syndrome characterized by aggressive, rapidly evolving ILD as recently confirmed (11, 50). Organizing pneumonia pattern with extensive, bilateral and consolidations at HRCT are typical of this subset, whereas signs of fibrosis are poorly represented (32), and pulmonary histology can show features of diffuse alveolar damage (33). Additional MSA are less frequently associated with the ILD onset, as in the case of anti-TIF1-gamma antibodies, which may be detected when malignancy coexists (34). A reduced risk of ILD has been reported with anti-NXP2 antibodies (28), but recent evidence has shown some inconsistency with this hypothesis (35). Indeed, a significant prevalence of NSIP and organizing pneumonia was reported in a cohort of anti-NXP2 positive patients, even if ILD tended to be clinically indolent (35). Anti-Mi-2 positivity is associated with a lower incidence of ILD, with good response to immunosuppressants, and favorable outcomes when compared to other forms of idiopathic inflammatory myopathies (36). The spectrum of immune-mediated necrotizing myopathies has been traditionally considered at low risk for extra-muscular manifestations, especially in anti-HMGCR positive cases associated with statin exposure (37). Nonetheless, recent data have suggested a significant prevalence of NSIP in patients with anti-SRP myositis, showing good treatment response and clinical stability throughout disease course (38). Antibodies directed at the nucleolar antigens PM/Scl-75 and PM/Scl-100 are frequently associated with late-onset, chronic NSIP in patients with PM/systemic sclerosis overlap (28, 39), and cases of isolated ILD have been reported in patients testing positive for such specificities (39). Anti-PM/Scl antibodies are found more often in patients with favorable outcomes (40), and no difference in survival was observed in a cohort of patients with anti-PM/Scl syndrome, irrespective of ILD (41). The PM/Scl-75 component is more frequently detected than the PM/Scl-100 (42) autoantigen, reported more frequently in association with a more active, inflammatory phenotype of myositis and ILD (39). Further evidence is required to demonstrate whether antibodies directed toward the two subunits are associated with different disease manifestations and might benefit from different therapies. Anti-Ku autoantibodies are rarely detected in patients with connective tissue diseases, and they can be associated with various clinical manifestations (43), including ILD especially when associated with myopathy (44) and in the absence of other detectable autoantibodies (45). While rare, the anti-Ku antibody is of outstanding importance when managing idiopathic inflammatory myopathy-ILD, since cases of resistance to corticosteroids and immunosuppressants have been reported (46).

**Table 1:** Association between myositis-specific and associated antibodies, the risk and clinical features of idiopathic inflammatory myopathy-ILD.

Autoantibody	Association with ILD	ILD pattern	Associated ILD features
Antisynthetase (28–31)	Strong	NSIP*, OP, UIP	Chronic, high mortality
MDA5 (11, 32, 33, 50)	Strong	OP*, NSIP	Rapidly-progressive, acute-subacute, refractory to therapy
PM/Scl (28, 39–42)	Strong	NSIP	Late onset, chronic, indolent
Ro52 (47–49)	Strong	Various	High predictor of ILD, poor outcomes if associated with anti-MDA5 and antisynthetase
NXP2 (28, 35)	Doubtful	NSIP, OP	Typically indolent
SRP (38)	Doubtful	NSIP	Good response to therapy
Ku (43–46)	Doubtful	Unknown	Refractory to therapy, impacts on prognosis
TIF1-gamma (34)	Weak	N.R.	N.R.
Mi-2 (36)	Weak	N.R.	N.R.
HMGCR (37)	Weak	N.R.	N.R.

*Most frequently observed pattern.

ILD, interstitial lung disease; N.R., not reported; NSIP, nonspecific interstitial pneumonia; OP, organizing pneumonia; UIP, usual interstitial pneumonia.

Anti-Ro52 antibodies still represent one of the most common autoantibodies in patients with connective tissue diseases (51, 52), with high prevalence of ILD with unfavorable outcomes (47–49). In particular cases, the coexistence of anti-Ro52 and anti-MDA5 antibodies has been associated with aggressive and rapidly progressive ILD in anti-MDA5 syndrome (53, 54) but there are conflicting data on the prognostic role of anti-Ro52 antibodies when associated with other MSA (55, 56). Of note, signs of lung fibrosis at HRCT were described in patients with ILD in mixed connective tissue disease and anti-Ro52 positivity (57), while lower prevalence of fibrosing ILD was found in a cohort of anti-Ro52 positive subjects with Sjogren’s syndrome compared to Ro52-negative patients (58). Remarkably, no autoantibody is currently able to predict PPF development in patients with idiopathic inflammatory myopathy-ILD. Given their prevalence and current clinical significance, elucidating the role of anti-Ro52 antibodies in this sense represents a major clinical unmet need.

### 3.1. Myositis autoantibodies associated with progressive pulmonary fibrosis in research and routine laboratories

There is currently no consensus on the autoantibody testing methodology beyond indirect immunofluorescence for antinuclear antibodies (ANA), generally the first-line for suspected connective tissue disease (59). In fact, autoantibodies in idiopathic inflammatory myopathies are associated with different staining patterns at indirect immunofluorescence (60) and ANA negativity has been reported in up to 50% of these patients in large cohorts (61, 62). Indeed, several myositis antigens (e.g., aminoacyl-tRNA-synthetases, MDA5, SRP) reside in the cytoplasm, and this can lead to false-negative ANA staining. However, indirect immunofluorescence is able to detect ANA suggestive of overlap syndromes, such as systemic sclerosis (63), and additional techniques such as immunoprecipitation still remain the gold standard to detect MSA and MAA. This method allows the testing of almost all known myositis antigens, analyzing antigens in their native conformation thus with highest sensitivity and specificity, and it is directed toward both protein and RNA components. Ultimately, immunoprecipitation provides conclusive evidence in most cases also for rare and uncommon autoantibodies (64) but the method is laborious, and expertise is required to perform it adequately. As a consequence, most diagnostics laboratories usually employ automated techniques, as immunoblot assays and enzyme-linked immunosorbent assays (ELISA), with variable sensitivity and specificity in the detection of several MSA and MAA (63), to screen for multiple antigens at once. The performance of myositis immunoblot might be inferior when compared to gold standard techniques (63) and multiple MSA positivities in single patients have been reported with the use of immunoblot (65) but results should be interpreted with caution. Combining ANA indirect immunofluorescence and immunoblot has been proposed to implement the diagnostic performance in patients with idiopathic inflammatory myopathies (66). Discrepancies between the antigen individuated with immunoblot and ANA staining pattern should orient toward a false-positive immunoblot result (67); when applied to an appropriate clinical context (as is the case of suspected idiopathic inflammatory myopathy-ILD), immunoblot can prove helpful (68). Other autoantibodies, such as anti-Ro52, are not detectable by immunoprecipitation and require specific changes in the immunoprecipitation assays protocol (69, 70). The serological discrimination of anti-Ro52 from anti-Ro60 antibodies is essential because they are associated with different clinical entities (71) thus overcoming the historical ‘anti-Ro/SSA’ denomination (without distinction between the two antigens) that should be abandoned (71).

## 4. Results of our monocentric study on anti-Ro52 in idiopathic inflammatory myopathy-ILD

We retrospectively analyzed a cohort of patients with idiopathic inflammatory myopathies, and described the main demographic, clinical, and serological features, focusing on the anti-Ro52 status. We also analyzed on patients with idiopathic inflammatory myopathy-ILD, comparing clinical, functional, radiological (HRCT), and serological characteristics. Serum immunoprecipiation for MSA/MAA was performed according to established methods (72) while anti-Ro52 antibodies were tested by ELISA.

Supplementary Table 1 illustrates the characteristics of the cohort of 55 patients with a diagnosis of idiopathic inflammatory myopathy included in the study. ANA at titer ≥1:160 were detected in 42/55 (76%) patients, and anti-Ro52 ELISA tested positive in 14/55 (25%) sera. Median ages at diagnosis were 52.5 years (range 38.5–60.5 years) in the anti-Ro52 negative group, and 48.5 years (range 45–62 years) in the anti-Ro52 positive group. No significant differences in the gender ratio, prevalence of malignancy and coexisting autoimmune disorders were observed between the two groups. ILD was significantly more prevalent in the anti-Ro52 positive group (79%) than in the anti-Ro52-negative group (37%; *p* = 0.007), while no difference was observed for other clinical manifestations such as myositis, skin rash, Raynaud phenomenon, arthritis, dysphagia, and cardiomyopathy. As for autoimmune serological results, antisynthetase antibodies occurred much more frequently in the Ro52-positive group, but no significant differences were reported for anti-MDA5, anti-PM/Scl, and other MSA/MAA status between the two groups.

Table 2 summarizes the main features of patients with idiopathic inflammatory myopathy-ILD based on their anti-Ro52 status. The Ro52-positive group was younger (median age 49 versus 55 years in Ro52-negative subjects), but no significant differences were retrieved in terms of demographic and clinical features (including baseline creatine kinase values), except for a predominance of Raynaud phenomenon in the Ro52-negative group. The two groups did not differ in terms of pulmonary function tests, as baseline values of forced vital capacity and diffusing capacity for carbon monoxide were similar and the proportion of patients with worsening pulmonary function was comparable. Imaging findings from baseline HRCT were analyzed for the detection of ground glass opacities, consolidations, and signs of fibrosis (defined as the presence of subpleural reticulation, traction bronchiectasis, and/or honeycombing) (18), and no differences were observed in terms of consolidations between the two groups. Remarkably, ground glass opacity was significantly more frequent in patients testing negative for anti-Ro52 antibodies and signs of fibrosis were more prevalent in patients with anti-Ro52 positivity (82%) than in negative subjects (30%, *p* = 0.0189). As for serological results, a higher prevalence of antisynthetase antibodies was confirmed in the Ro52-positive group, also when considering the ILD subgroup, while no differences were found for ANA, anti-MDA5, anti-PM/Scl, and other MSA/MAA.

**Table 2:** Demographic, clinical, autoimmune features of the studied cohort of patients with inflammatory myositis and ILD, based on their anti-Ro52 status.

	Ro52 positive (*n* = 11)	Ro52 negative (*n* = 15)	*p*
Age, years (range)	49 (45–71)	55 (49–63)	–
Female sex	10 (91)	11 (73)	0.2607
Malignancy	3 (27)	1 (7)	0.1718
Overlap AID	3 (27)	4 (27)	1.0000
Myositis	8 (73)	12 (80)	0.6810
Skin rash (DM)	9 (82)	12 (80)	0.9001
Raynaud’s phenomenon	3 (27)	10 (67)	0.0481
Capillaroscopy alterations	7 (64)	9 (60)	0.8389
Arthritis	5 (45)	5 (33)	0.5416
Cardiomyopathy	2 (18)	4 (27)	0.5984
Dysphagia	2 (18)	6 (40)	0.2387
Basal FVC	93 (82–102)	95 (84–105)	0.8259
FVC decline >5% over 1 year	4/6 (67)	3/7 (43)	0.4056
Basal DLCO	67.5 (59–79)	67 (54–76)	0.6527
DLCO decline >10% over 1 year	1/6 (17)	1/7 (14)	0.8858
Ground glass opacity	4 (36)	8/10 (80)	0.0472
Consolidations	1 (9)	2/10 (20)	0.4820
Signs of fibrosis	9 (82)	3/10 (30)	0.0189
Elevated baseline CPK	5 (45)	12 (80)	0.0695
ANA ≥1:160	10 (91)	12 (80)	0.4509
Antisynthetase antibodies	7 (64)	2 (13)	0.0081
Anti-MDA5	0 (0)	1 (7)	0.3797
Anti-PM/Scl	1 (9)	3 (20)	0.4509
Other MSA/MAA	1 (9) (TIF1-gamma)	3 (20) (TIF1-gamma, SAE, RNP)	0.4509

AID, autoimmune disease (i.e., thyroiditis, psoriasis, coeliac disease, lichen planus, systemic lupus erythematosus, autoimmune hepatitis, rheumatoid arthritis, autoimmune gastritis); ANA, antinuclear antibodies at a titer ≥ 1:160; CPK, creatine phosphokinase; DLCO, diffusing capacity for carbon monoxide; DM, dermatomyositis; FVC, forced vital capacity; GGO, ground glass opacities; HRCT, high-resolution CT scan; ILD, interstitial lung disease; MAA, myositis-associated antibodies; MSA: myositis-specific antibodies; PFT: pulmonary function tests.

### 4.1. Data interpretation

As shown in our monocentric analysis on anti-Ro52 patients with idiopathic inflammatory myopathy-ILD, anti-Ro52 antibodies are strong predictors of ILD development, significantly associated with antisynthetase antibodies, as confirmed by previous findings (52, 56). We extensively describe the association between anti-Ro52 positivity and signs of lung fibrosis at HRCT in a cohort of patients with idiopathic inflammatory myopathy-ILD, similar results were achieved for mixed connective tissue disease-ILD (57). It should be kept in mind that fibrosing signs at HRCT represent the risk for PPF in patients with connective tissue disease-ILD (23), and antifibrotic therapy is now advised when PPF develops (18). In our cohort, anti-Ro52 antibodies were not associated with a functional decline of lung capacity over 1 year of observation, but we are aware that this might be due to the small sample size and to the short period of observation. Finally, the presence of ground glass opacity was negatively correlated with anti-Ro52 status, suggesting that anti-Ro52 might play a role in more chronic, insidious, fibrosing processes than in acute/subacute subtypes.

Ro52/TRIM21 is a E3-ubiquitin ligase owing to the TRIM superfamily and several members of this superfamily are involved in fibrosing processes, including lung fibrosis (73). TRIM21 interacts with TGF-beta expression and function (73), and regulates the inflammatory response, e.g., balancing the pro-inflammatory effects of NF-kB (74). Pirfenidone is an antifibrotic drug currently approved for the treatment of idiopathic pulmonary fibrosis (75) and the drug acts by down regulating pro-fibrotic signaling pathways, molecules, and cells, although precise molecular mechanisms are still to be explored (76). TRIM21 expression in idiopathic pulmonary fibrosis lung fibroblasts is regulated by pirfenidone (77), and Ro52/TRIM21 activity might be correlated with lung fibrosis in patients with idiopathic pulmonary fibrosis. These aspects may be applicable to other forms of PPF, considering the crucial role of TRIM proteins in the pathogenesis of fibrosis. Anti-Ro52 antibodies correlate with lung fibrosis at HRCT and, thus, they could represent a risk factor for PPF, especially in case of idiopathic inflammatory myopathy-ILD. Further studies are required to support the hypothesis of increased risk of lung fibrosis and PPF in anti-Ro52 positive patients with idiopathic inflammatory myopathies myositis-ILD, and to elucidate the possible role of Ro52/TRIM21 in the pathogenesis of lung fibrosis. A potential role of pirfenidone therapy in patients with progressive fibrosing idiopathic inflammatory myopathy-ILD might be hypothesized, especially in case of anti-Ro52 positivity. Antifibrotic therapy has changed the course and prognosis of idiopathic pulmonary fibrosis, and similar results are expected in patients with PPF, including cases of idiopathic inflammatory myopathy-ILD. A precision medicine approach, based on the correct autoantibody determination, is required to offer targeted immunosuppressive and antifibrotic therapies to patients with idiopathic inflammatory myopathy-ILD.

## 5. Conclusion

It is crucial to screen for idiopathic inflammatory myopathies in patients with ILD and a cluster of myositis autoantibodies is significantly associated with ILD onset in these patients. Currently, there is no established risk factor for PPF in patients with idiopathic inflammatory myopathy, and serum autoantibodies are ideal candidates in this sense. We report and discuss the implications of the association between anti-Ro52 antibodies and lung fibrosis in a cohort of patients with idiopathic inflammatory myopathies, and we speculate that anti-Ro52 may represent a risk factor for PPF in these patients. Data from larger cohorts and longer follow-up periods are required to corroborate this hypothesis. Other myositis autoantibodies should be also tested.

## Data availability statement

The data that support the findings of this study are available from the corresponding author, CS, upon reasonable request.

## Ethics statement

The studies involving human participants were reviewed and approved by Humanitas Research Hospital. The patients/participants provided their written informed consent to participate in this study.

## Author contributions

All authors listed have made a substantial, direct, and intellectual contribution to the work and approved it for publication.

## Funding

This work was partially supported by “Ricerca Corrente” funding from Italian Ministry of Health to IRCCS Humanitas Research Hospital.

## Conflict of interest

The authors declare that the research was conducted in the absence of any commercial or financial relationships that could be construed as a potential conflict of interest.

The reviewer GDL declared a past co-authorship with the author(s) MDS to the handling editor.

## Publisher’s note

All claims expressed in this article are solely those of the authors and do not necessarily represent those of their affiliated organizations, or those of the publisher, the editors and the reviewers. Any product that may be evaluated in this article, or claim that may be made by its manufacturer, is not guaranteed or endorsed by the publisher.
