# Supplementary material for: Interstitial lung disease associated with inflammatory myositis: Autoantibodies, clinical phenotypes, and progressive fibrosis
Source: Front Med (Lausanne). 2023 Mar 16;10:1068402. doi: 10.3389/fmed.2023.1068402 (PMC10061022; doi:10.3389/fmed.2023.1068402)
Supplement: Supplementary file 1 [file Table_1.pdf]

Supplementary Table. Demographic, clinical, autoimmune features of a cohort of patients with inflammatory myositis, based on their anti-Ro52 status.

	**Ro52 positive (n = 14)**	**Ro52 negative (n = 41)**	***p***
Female sex	13 (93)	30 (73)	.1217
Malignancy	4 (29)	9 (22)	.5987
Overlap AID	4 (29)	10 (24)	.7127
Myositis	10 (71)	32 (78)	.5987
Skin rash (DM)	10 (71)	28 (68)	.8358
Raynaud's phenomenon	5 (36)	18 (44)	.6038
Capillaroscopy alterations	9 (64)	21 (51)	.4035
Arthritis	5 (36)	9 (22)	.3043
Cardiomyopathy	2 (14)	6 (15)	.9281
Dysphagia	2 (14)	10 (24)	.4355
ILD	11 (79)	15 (37)	**.0071**
Elevated baseline CPK	7 (50)	28 (68)	.2316
ANA ≥ 1:160	13 (93)	29 (71)	.0962
Antisynthetase antibodies	7 (50)	2 (5)	**.0001**
Anti-MDA5	0 (0)	2 (5)	.3982
Anti-PM/Scl	1 (7)	4 (10)	.7401
Other MSA/MAA	2 (14)	9 (22)	.5218

List of abbreviations – AID: autoimmune disease (i.e. thyroiditis, psoriasis, coeliac disease, lichen planus, systemic lupus erythematosus, autoimmune hepatitis, rheumatoid arthritis, autoimmune gastritis); ANA: antinuclear antibodies at a titer ≥ 1:160; CPK: creatine phosphokinase; DM: dermatomyositis; ILD: interstitial lung disease; MAA: myositis-associated antibodies; MSA: myositis-specific antibodies.
